# Supplementary material for: Burnout in Pediatric Oncology: Team Building and Clay Therapy as a Strategy to Improve Emotional Climate and Group Dynamics in a Nursing Staff
Source: Cancers (Basel). 2025 Mar 25;17(7):1099. doi: 10.3390/cancers17071099 (PMC11988093; doi:10.3390/cancers17071099)
Supplement: Supplementary file 1 [file cancers-17-01099-s001.zip › cancers-3506982-supplementary.pdf]

Supplementary Materials

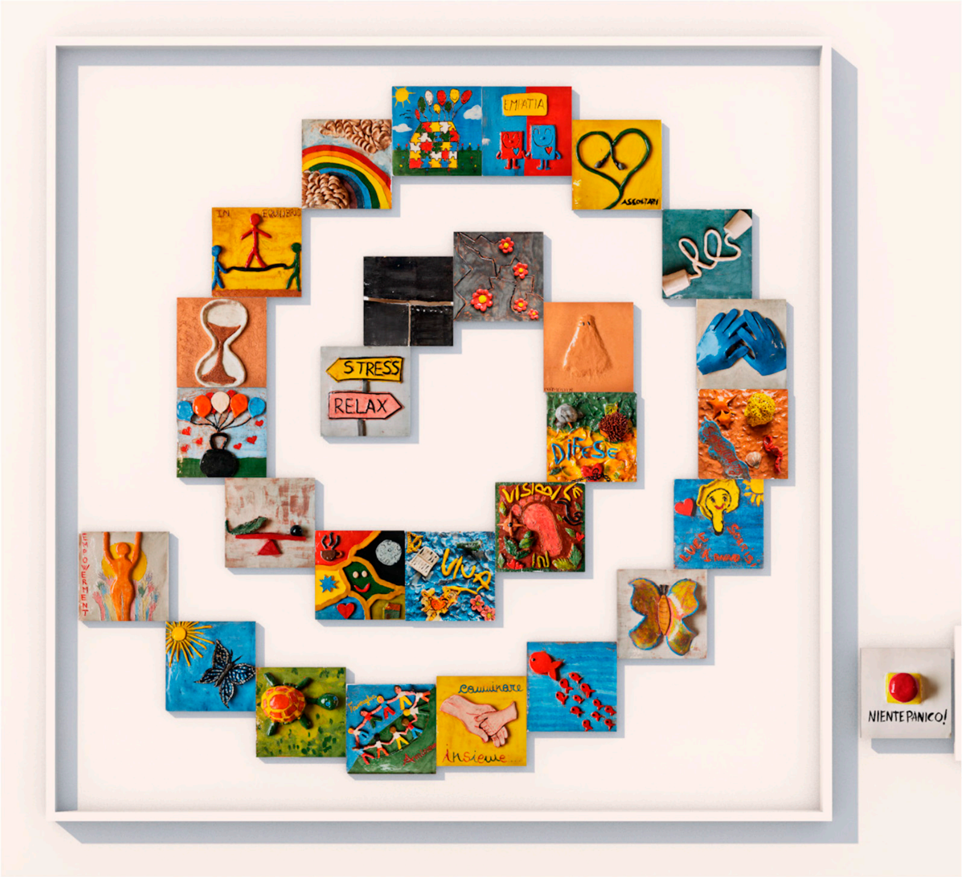

Figure S1. The concluding artistic work "The Burnout Spiral".

Caption:

Burn out: a spiral in motion

"ArtOut" is the need to make visible, through the plastic material of clay, the thoughts, experiences and complex emotions, with the aim of cushioning and preventing the effects of "Burn-out" (work stress). This work is the result of a path creative, individual and collective, aimed at improve the working climate of the staff. The tiles make up a spiral that from the center seems to expand outward outward, or from the outside return to the center, but which is always the image of a movement: courageous, necessary and vital. The spiral is one of the most ancient and fascinating precisely because of its ability to reconcile opposites: introspection and expansion, involution and evolution; it is a vortex that sucks in but also energy that radiates. And in this complexity, as the 28th tile suggests:"don't panic!"
